# PGC‐1α is downregulated in a mouse model of obstructive cholestasis but not in a model of liver fibrosis

**DOI:** 10.1002/2211-5463.12961

**Published:** 2020-12-09

**Authors:** Jung Hyun Park, Bong Jun Kwak, Ho Joong Choi, Ok‐Hee Kim, Ha‐Eun Hong, Sang Chul Lee, Kee‐Hwan Kim, Young Kyoung You, Tae Yun Lee, Joseph Ahn, Say‐June Kim

**Affiliations:** ^1^ Department of Surgery College of Medicine Eunpyeong St. Mary's Hospital the Catholic University of Korea Seoul Korea; ^2^ Department of Surgery College of Medicine Incheon St. Mary's Hospital The Catholic University of Korea Incheon Korea; ^3^ Department of Surgery College of Medicine Seoul St. Mary’s Hospital the Catholic University of Korea Seoul Korea; ^4^ Catholic Central Laboratory of Surgery Institute of Biomedical Industry College of Medicine the Catholic University of Korea Seoul Korea; ^5^ Department of Surgery College of Medicine Daejeon St. Mary's Hospital the Catholic University of Korea Daejeon Korea; ^6^ Department of Surgery College of Medicine Uijeongbu St. Mary's Hospital the Catholic University of Korea Gyeonggi‐do Korea

**Keywords:** antioxidant enzymes, cholestatic liver disease, liver fibrosis, mitochondria, peroxisome proliferator‐activated receptor‐γ co‐activator 1α

## Abstract

Several studies have indicated that cholestatic liver damage involves mitochondria dysfunction. However, the precise mechanism by which hydrophobic bile salts cause mitochondrial dysfunction is not clear. In this study, we intended to determine the pathogenesis of cholestatic liver injury associated with peroxisome proliferator‐activated receptor‐γ co‐activator 1α (PGC‐1α). A mouse model of cholestatic liver disease was generated by surgical ligation of the bile duct (BDL), and a mouse model of fibrosis was developed through serial administration of thioacetamide. After obtaining liver specimens on scheduled days, we compared the expression of the antioxidant enzymes (superoxide dismutase 2 [SOD2], catalase, and glutathione peroxidase‐1[GPx‐1]) and PGC‐1α in livers from mice with fibrosis and cholestasis using western blotting, immunohistochemistry, and immunofluorescence. We found that cholestatic livers exhibit lower expression of antioxidant enzymes, such as SOD2, catalase, and PGC‐1α. In contrast, fibrotic livers exhibit higher expression of antioxidant enzymes and PGC‐1α. In addition, cholestatic livers exhibited significantly lower expression of pro‐apoptotic markers (Bax) as compared to fibrotic livers. It is well known that overexpression of PGC‐1α increases mitochondrial antioxidant enzyme expression, and vice versa. Thus, we concluded that obstructive cholestasis decreases expression of PGC‐1α, which may lead to decreased expression of mitochondrial antioxidant enzymes, thereby rendering mice with cholestatic livers vulnerable to ROS‐induced cell death.

## Graphical abstract

Herein, we determined the pathogenesis of cholestatic liver injury in relation to peroxisome proliferator‐activated receptor‐γ co‐activator 1α (PGC‐1α), a potent regulator of energy metabolism and mitochondrial biogenesis. We validated that obstructive cholestasis decreases expression of PGC‐1α, which leads to decreased expression of mitochondrial antioxidant enzymes, thereby rendering mice with cholestatic livers vulnerable to reactive oxygen species (ROS)‐induced cell death.

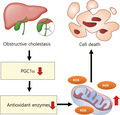

**Abbreviations**
Bax: Bcl‐2‐like protein 4
BDL: bile duct ligation
FFPE: formalin‐fixed, paraffin‐embedded
GDCAs: hydrophobic bile salts
NRF‐1: nuclear respiratory factors
PGC‐1α: peroxisome proliferator‐activated receptor‐γ co‐activator 1α
SOD: superoxide dismutase 2
TAA: thioacetamide

Obstructive cholestasis is defined as decrease in bile flow due to its obstruction through intra‐ or extrahepatic bile ducts. Obstructive cholestasis is a frequently occurring disease. Any diseases that cause narrowing of the biliary duct, frequently the extrahepatic bile duct, can induce obstructive cholestasis, including cholangitis, cholelithiasis, pancreatic cancer, duodenal cancer, and ampulla of Vater cancer. Physiologically, bile salts are recycled within a circuit of enterohepatic circulation; they are first synthesized in the liver; then, they enter the intestine by way of the biliary tract and finally return to the liver through the portal vein after being reabsorbed in the proximal and distal ileum. As obstructive cholestasis is characterized by the abruption of such an enterohepatic circulation, it has systemic impacts, including decreased protein synthesis in the liver, decreased gut mucosal integrity, increased bacterial translocation, decreased myocardial contractibility, and systemic vasodilatation [1, 2].

Of those organs, the liver is a direct target of obstructive cholestasis because of the bile salts accumulated in the liver. Currently, several studies indicate that hepatotoxicity by hydrophobic bile salts is mitochondria‐mediated [3, 4, 5, 6]. It was revealed that hydrophobic bile acids induce alterations in membrane fluidity, which is associated with impairment of mitochondrial respiration and mitochondrial depolarization [5]. However, little has been known about the precise mechanism by which hydrophobic bile salts cause mitochondrial dysfunction. In this study, we focused on the function of peroxisome proliferator‐activated receptor‐γ co‐activator 1α (PGC‐1α). PGC‐1α is a potent regulator of energy metabolism and mitochondrial biogenesis [7, 8, 9, 10]. As a regulator of mitochondrial biogenesis and function, PGC‐1α binds to a variety of transcriptional factors, including mitochondrial DNA transcription factor A, transcriptional nuclear respiratory factors (NRF‐1), and other metabolic transcriptional nuclear factors [10]. PGC‐1α is also known to regulate the expression and activity of mitochondrial antioxidant enzymes in various cells [11, 12]. Overexpression of PGC‐1α leads to upregulation of mitochondrial antioxidant enzymes and to the downregulation of oxidative stress in vascular endothelial cells [11]. In this study, we intended to determine the pathogenesis of hepatotoxicity caused by obstructive cholestasis associated with PGC‐1α.

## Materials and methods

### In vitro experiments

#### Chemicals

Hydrophobic bile salts (GDCAs) were purchased from Sigma‐Aldrich (St Louis, MO, USA). 5,5′,6,6′‐tetrachloro‐1,1′,3,3′‐tetraethylbenzimidazolcarbocyanine iodide (JC‐1) kit was purchased from Sigma‐Aldrich (St Louis).

#### Cell culture

The LO2 human hepatocyte cell lines were kindly donated by D‐H Kim (Konkuk University, South Korea). The LO2 hepatocyte cell lines were maintained in DMEM high‐glucose medium (Thermo, Carlsbad, CA, USA). The medium was supplemented with 10% fetal bovine serum (FBS, Gibco‐BRL, Carlsbad, CA, USA) and 1% antibiotics (Thermo) at 37 °C in a humidified atmosphere with 5% CO_2_ in an incubator.

#### Overexpression of PGC‐1α genes

pcDNA3.1‐PGC‐1α was purchased from Addgene (Watertown, MA, USA). Briefly, LO2 cells were plated in 6‐well plates (2 × 10^5^ cells/well) and transiently transfected with 100 nm per well of pcDNA3.1‐PGC‐1alpha mixed with the Lipofectamine 2000 transfection reagent (Thermo Fisher Scientific) according to the manufacturer’s instructions. pcDNA3.1 vector (Addgene, Watertown, MA, USA) was used as a negative control and introduced into the cells under the same protocol. Transcription was specifically suppressed by siRNA, which targeted PGC‐1 mRNA coding sequence. Briefly, LO2 cells were plated in 6‐well plates (2 × 10^5^ cells/well) and transiently transfected with 100 nm per well of PGC‐1 siRNA (Santa Cruz Biotechnology, Santa Cruz, CA, USA) mixed with the Lipofectamine RNAiMAX transfection reagent (Thermo Fisher Scientific) according to the manufacturer’s instructions. Silencer Negative Control siRNA (Santa Cruz) was used as a negative control and introduced into the cells under the same protocol. After 5‐h incubation, the medium was changed to complete culture medium, and the cells were incubated at 37 °C in a CO_2_ incubator for 48 h before harvesting.

#### JC‐1 mitochondrial membrane potential assay

LO2 cells were plated in 6‐well plates (2 × 10^5^ cells/well) and transiently transfected with 1 μg pcDNA3.1‐PGC‐1α and 100 nm PGC‐1α siRNA. After transfection for 24 h, LO2 cells were cultured in the medium with 100 μm GDCA for 24 h. After that, LO2 cells were stained with 5 μm JC‐1 for 15 min at 37 °C in the dark. Fluorescence was observed under an EVOS M5000 fluorescence microscope (Thermo Fisher Scientific).

### *In vivo* experimental design

#### Mouse model of obstructive cholestasis

Six‐week‐old male BALB/c mice (OrientBio Animal Center, Seongnam, Republic of Korea) (*N* = 70) were used for this study. This study was approved by the Institutional Animal Care and Use Committee of the Clinical Research Institute at Seoul St. Mary’s Hospital at the Catholic University of Korea (CUMC‐2018‐0202‐01). Mice were anesthetized using an intraperitoneal injection of 30 mg/kg^−1^ tiletamine–zolazepam (Zoletil 20; Virbac, Nice, France). We generated a mouse model of obstructive cholestasis by bile duct ligation (BDL) as previously described [13]. Briefly, after opening the abdomen by performing a midline laparotomy, we exposed the bile duct by caudal movement of the gut. We then sutured the bile duct using the 4‐0 silk suture and secured it with two surgical knots. Before obtaining the liver specimens, mice were fasted overnight to minimize variability due to dietary uptake. To minimize the effects of the circadian rhythm, we euthanized the mice at the fixed time (10 am to 12 pm) and obtained liver and blood samples. All manipulations were performed at 4 °C or on ice to minimize mitochondrial membrane and protein degradation. The intervals of collecting liver specimens were 12 h, and 0, 1, 2, 3, 4, 7, and 14 days, respectively, after BDL (*N* = 10 per day). Collected liver tissues were processed for protein isolation, and the rest of the tissues were fixed for pathology or stored at − 80 °C.

#### Mouse model of TAA‐induced liver fibrosis

Six‐week‐old male BALB/c mice (*N* = 20) were used for this study. Hepatic fibrosis was induced in these mice using an intraperitoneal injection of thioacetamide (TAA) (Sigma, St Louis, MO, USA: 200 mg/kg body weight) two times a week for the determined periods (3, 5, and 8 weeks), respectively. One week after the last injection of TAA, mice were euthanized, and their liver specimens were collected for investigation. Before obtaining the liver specimens, mice were fasted overnight. All manipulations were performed at 4 °C or on ice at the fixed time (10 am to 12 am).

#### Immunohistochemical analyses and trichrome staining

For immunohistochemical analysis, formalin‐fixed, paraffin‐embedded (FFPE) tissue sections were deparaffinized, rehydrated in an ethanol series, and subjected to epitope retrieval using standard procedures. Antibodies against PGC‐1α, superoxide dismutase 2 (SOD2), and Bcl‐2‐like protein 4 (Bax) (all from Cell Signaling Technology, Danvers, MA, USA); cytochrome c (Santa Cruz, Dallas, TX); 4‐hydroxy‐2‐nonenal (4‐HNE; Abcam, Cambridge, UK); and 8‐hydroxy‐2'‐deoxyguanosine (8‐OHdG; Abcam) were used for immunochemical staining. The samples were then examined under a laser scanning microscope (Eclipse TE300; Nikon, Tokyo, Japan) to analyze the expression of PGC‐1α, SOD2, and Bax. Trichrome staining was performed using the Masson’s trichrome staining kit according to the manufacturer’s protocol (Polysciences, Warrington, PA, USA).

#### Biochemical analysis

Mice were fasted overnight before obtaining blood samples. Serum was collected from mouse blood samples and was centrifuged for 10 min at 10 000 ***g***. Serum concentrations of biochemical parameters indicative of liver injury, including aspartate transaminase (AST), alanine transaminase (ALT), ammonia and, and total bilirubin, were determined using a VetTest Chemistry Analyzer (IDEXX Laboratories; Westbrook, ME, USA).

#### Western blotting

Liver tissues were lysed using the EzRIPA Lysis Kit (ATTO Corporation; Tokyo, Japan) and quantified using the Bradford reagent (Bio‐Rad, Hercules, CA, USA). Proteins were visualized using western blotting with the following primary antibodies (1 : 1000 dilution) at 4 °C overnight and with HRP‐conjugated secondary antibodies (1 : 2000 dilution) for 1 h at 25 °C. Primary antibodies included antibodies against PGC‐1α, SOD2, catalase, glutathione peroxidase‐1 (GPx‐1), B‐cell lymphoma 2 (Bcl‐2), and β‐actin (all from Cell Signaling Technology). Secondary antibodies were horseradish peroxidase (HRP)‐conjugated (Cell Signaling Technology). Specific immune complexes were detected using the Western Blotting Plus Chemiluminescence Reagent (Millipore; Bedford, MA, USA).

### Statistical analysis

All data were analyzed using the SPSS 11.0 software (SPSS Inc.; Chicago, IL, USA) and are presented as the mean ± standard deviation (SD). Statistical comparisons between the mean values of two groups were performed using Mann–Whitney *U*‐test; for the comparison of three or more groups, the Kruskal–Wallis test was used. Probability (*P*) values of < 0.05 were considered statistically significant.

## Results

### Effects of hydrophobic bile salts on the expression of PGC‐1α and antioxidant enzymes

**Fig. 1:**
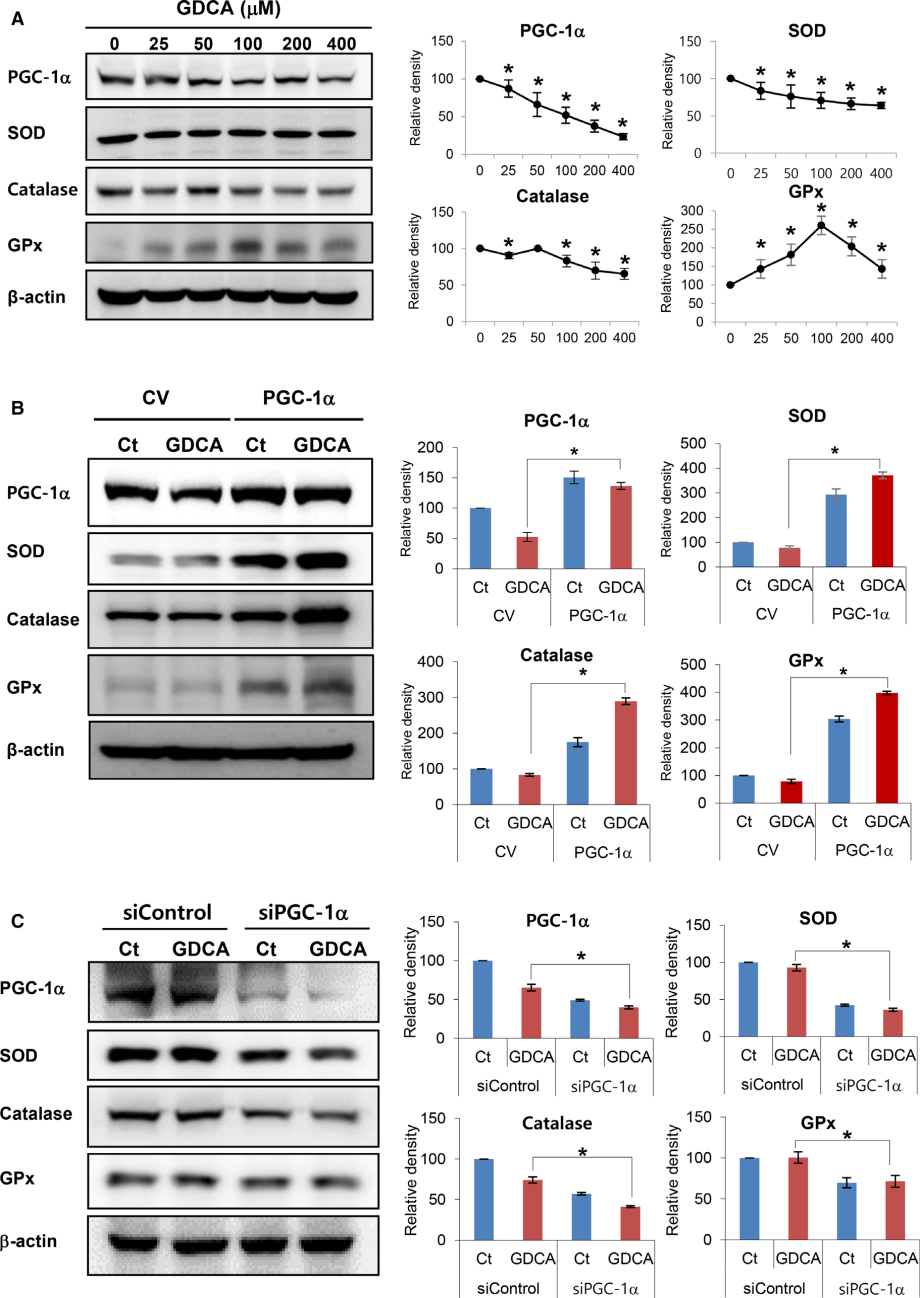
Effects of hydrophobic bile salts on the expression of PGC‐1α and antioxidant enzymes. (A) Western blot analysis showing the direct effect of hydrophobic bile salts (GDCAs; hydrophobic bile salts) on the hepatocytes. GDCAs dose‐dependently decreased the expression of PGC‐1α. GDCAs also dose‐dependently decreased the expression of SOD2 and catalase, and decreased the expression of GPx‐1 after temporal elevation of the expression. (B) Western blot analysis showing the effect of overexpressing PGC‐1α on the expression of antioxidant enzymes. Overexpression of PGC‐1α abrogated the effects of reducing the expression of antioxidant enzymes by GDCAs. (C) Western blot analysis showing the effect of downregulating PGC‐1α on the expression of antioxidant enzymes. Downregulation of PGC‐1α further potentiated the effects of reducing the expression of antioxidant enzymes by GDCAs. Relative densities of individual markers had been quantified using ImageJ software and then were normalized to those of β‐actin in each group. Mann–Whitney *U*‐test was used for two groups and Kruskal–Wallis test for three or more groups. Values are presented as mean ± standard deviation of three independent experiments. *P < 0.05. Abbreviations: Bax, Bcl‐2‐like protein 4; Ct, control; GDCAs, hydrophobic bile salts; GPx‐1α, glutathione peroxidase‐1 alpha; PGC‐1α, peroxisome proliferator‐activated receptor‐γ co‐activator 1α; siControl, siRNA control; SOD2, superoxide dismutase 2

We first investigated the direct effect of hydrophobic bile salts on the hepatocytes. LO2 normal hepatocytes were treated with the increasing concentration of hydrophobic bile salts (GDCAs; hydrophobic bile salts), and subsequently, the alterations in the protein expression of the LO2 cells were determined by western blot analysis (Fig. 1A). GDCAs dose‐dependently decreased the expression of PGC‐1α. In addition, GDCAs dose‐dependently decreased the expression of SOD2 and catalase, and decreased the expression of GPx‐1 after temporal elevation. Next, we investigated alterations in the expression of antioxidant enzymes in LO2 cells when PGC‐1α was overexpressed. Overexpression of PGC‐1α abrogated the effects of reducing the expression of antioxidant enzymes, such as SOD2, catalase, and GPx‐1, by GDCAs (Fig. 1B). Subsequently, we investigated alterations in the expression of antioxidant enzymes in LO2 cells when PGC‐1α was downregulated by siPGC‐1α. Downregulation of PGC‐1α further potentiated the effects of reducing the expression of antioxidant enzymes by GDCAs (Fig. 1C). Taken altogether, the above results indicate that PGC‐1α could act as a regulator in the expression of antioxidant enzymes by GDCA.

### Determination of the role of PGC‐1α during the alteration of mitochondrial transmembrane potential by GDCAs

**Fig. 2:**
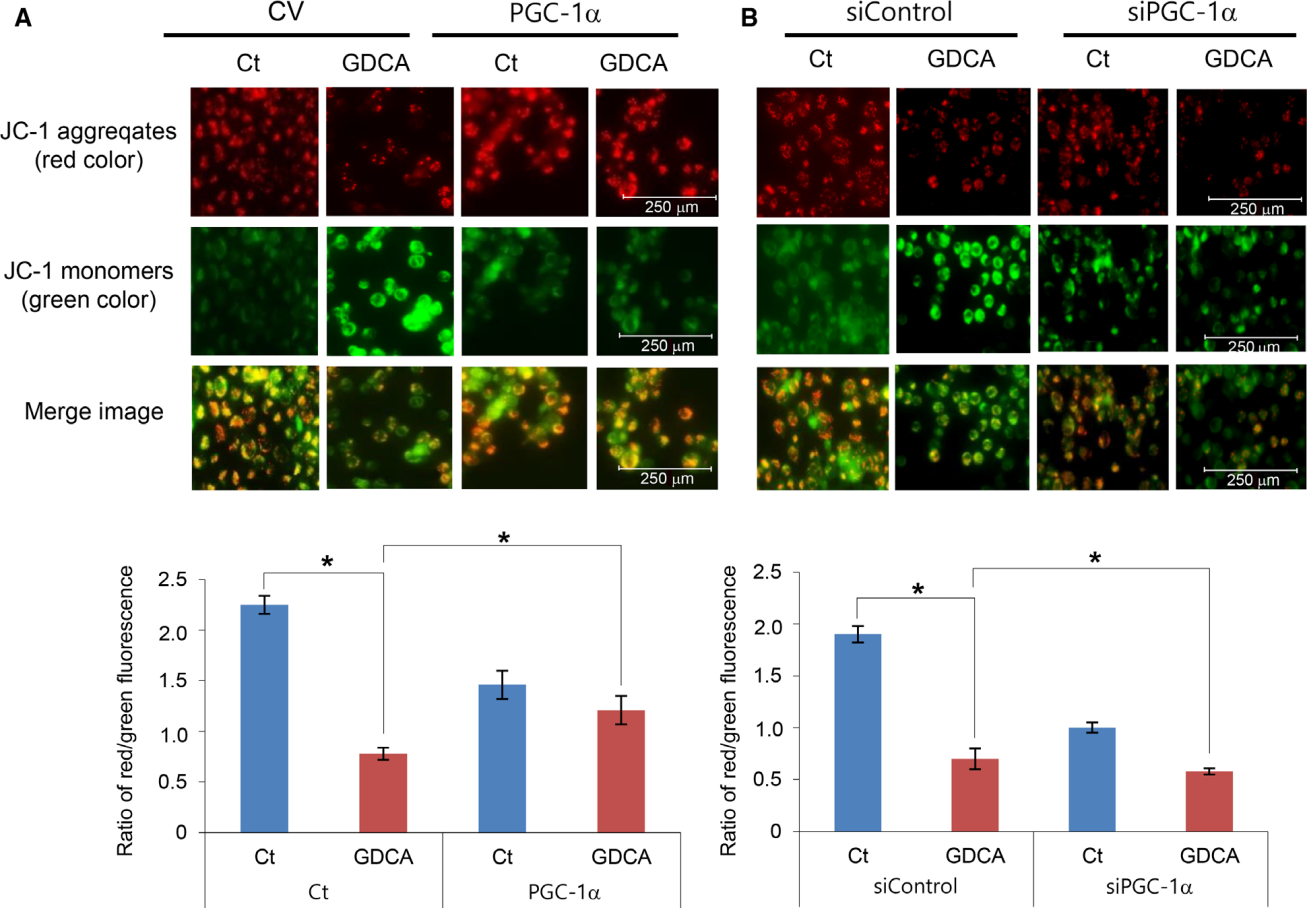
Determination of the role of PGC‐1α during the alteration of mitochondrial transmembrane potential by GDCAs. (A) JC‐1 assays to determine the effects of GDCAs on the mitochondrial membrane potential (MMP) in the PGC‐1α‐overexpressing LO2 cells. PGC‐1α overexpression significantly increased the ratio of red/green fluorescence that had been reduced by GDCAs (scale bar: 250μm). (B) JC‐1 assays to determine the effects of GDCAs on the MMP in the PGC‐1α‐downregulated LO2 cells. PGC‐1α downregulation further decreased the ratio of red/green fluorescence that had been reduced by GDCAs. The ratio of fluorescence intensity was measured using NIH ImageJ. Statistical comparison was performed using Kruskal–Wallis test (scale bar: 250 μm). Values are presented as mean ± standard deviation of three independent experiments. *P < 0.05. Abbreviations: Ct, control; GDCAs, hydrophobic bile salts; PGC‐1α, peroxisome proliferator‐activated receptor‐γ co‐activator 1α; siControl, siRNA control.

We performed JC‐1 assays to determine the effects of GDCAs on the mitochondrial membrane potential (MMP) in LO2 cells. GDCAs significantly increased green fluorescence emissions, which means that GDCAs impaired MMP, resulting in increased cellular apoptosis. Subsequently, we overexpressed PGC‐1α by transfecting pcDNA‐PGC‐1α into LO2 cells. PGC‐1α overexpression significantly increased the ratio of red/green fluorescence that had been reduced by GDCAs, suggesting PGC‐1α has the potential of recovering MMP that had been altered by GDCAs (Fig. 2A). Next, we performed additional JC‐1 assays to determine the effects of GDCAs on the MMP in the PGC‐1α‐downregulated LO2 cells. PGC‐1α‐downregulated LO2 cells were established by transfecting LO2 cells with siPGC‐1α. It was found that PGC‐1α downregulation further decreased the ratio of red/green fluorescence that had been reduced by GDCAs. Taken altogether, these results suggest that PGC‐1α has the potential of recovering MMP that had been altered by GDCAs (Fig. 2B).

### Establishment of the mouse model of obstructive cholestasis

**Fig. 3:**
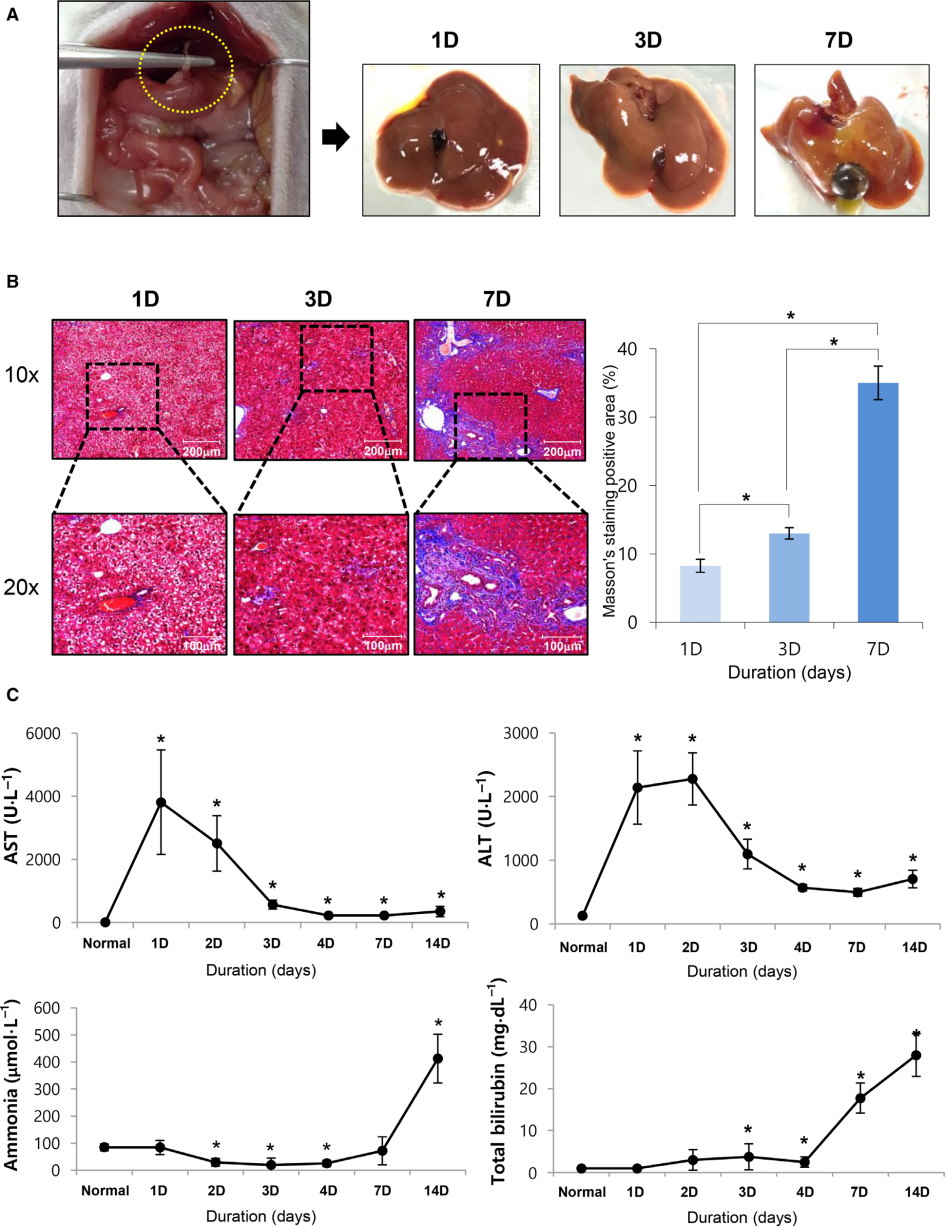
Establishment of the mouse model of obstructive cholestasis. (A) Generation of the mouse model of obstructive cholestasis. The mouse model of obstructive cholestasis was generated by surgical ligation of the bile duct. Prominent macronodularity of the liver was found on the 7th day after the procedure. (B) Liver specimens stained with Masson’s trichrome. The areas stained with Masson’s trichrome appeared to be significantly increased on 7 days, when they were compared to the areas on days 1 or 3. The percentage of fibrotic area were measured using NIH ImageJ. Statistical comparison was performed using Kruskal–Wallis test [scale bar, 200 μm (upper panels) and 100 μm (lower panels)]. (C) Biochemical parameters reflecting hepatic function after bile duct ligation (BDL). Of serum biochemical parameters, AST and ALT levels peaked 1‐2 days after the procedure, and ammonia and total bilirubin levels abruptly increased after days 7 and 4, respectively. Statistical comparison was performed using Mann–Whitney *U*‐test. Values are presented as mean ± standard deviation of three independent experiments. *P < 0.05. Abbreviations: ALT, alanine transaminase; AST, aspartate transaminase.

We developed a mouse model of obstructive cholestasis (*N* = 70) by surgically ligating the bile duct as described in the Methods section. On day 7 after the procedure, the size of the liver was reduced, and macronodularity across the surface of the liver became prominent (Fig. 3A). Next, we determined the degree of liver fibrosis by measuring the area stained with Masson’s trichrome in the liver specimens. The stained areas appeared to be significantly increased in the liver specimens on day 7 when they were compared to the liver specimens on day 1 or 3 (Fig. 3B). Subsequently, we consecutively assessed serum biochemical parameters reflecting hepatic function over time (Fig. 3C). Of the markers, AST and ALT levels peaked on 1‐2 days after the procedure, and those of ammonia and total bilirubin abruptly increased after 7 and 4 days, respectively.

### Effects of BDL on the expression of antioxidant enzymes in the liver

**Fig. 4:**
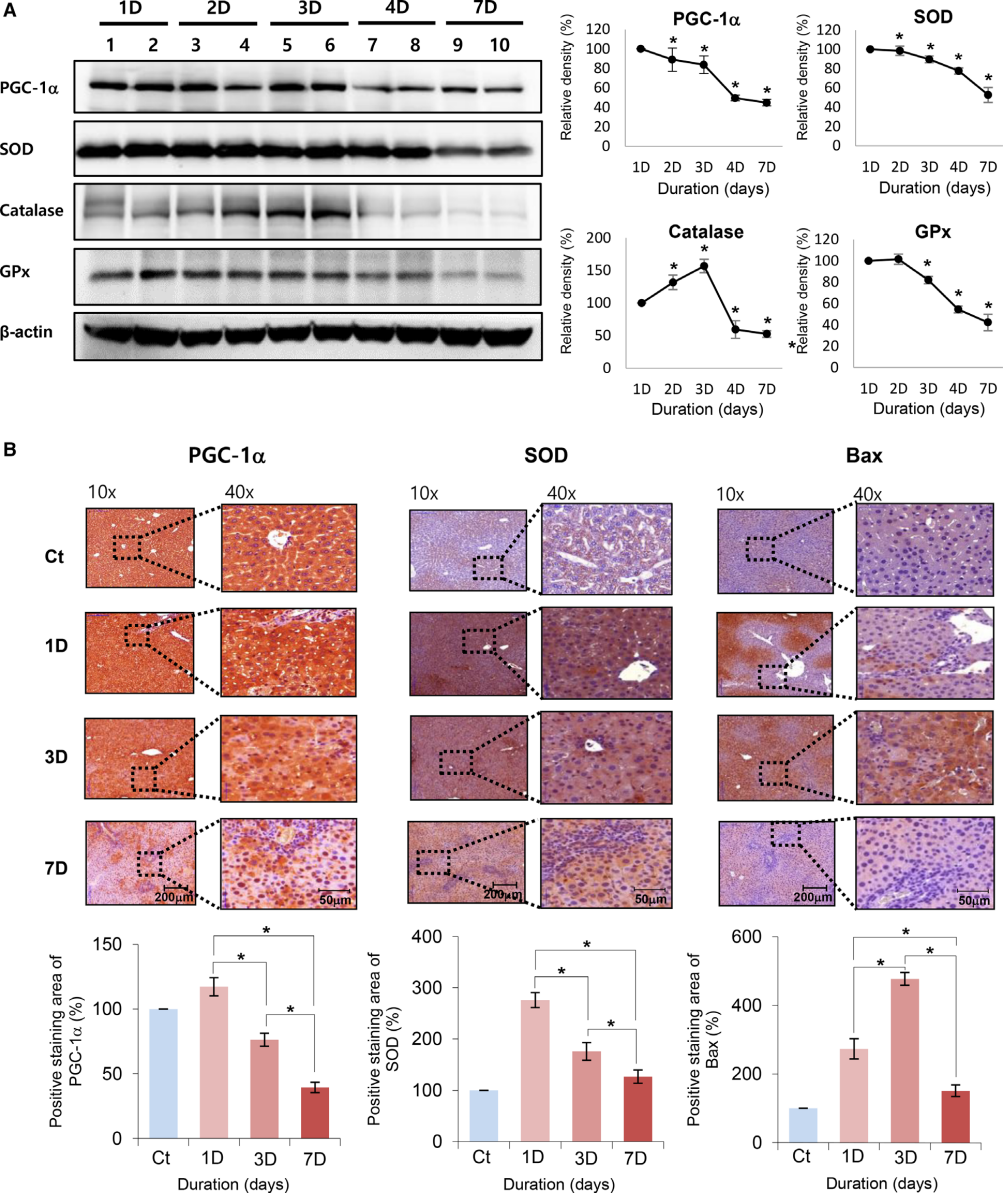
Effects of obstructive cholestasis on the expression of PGC‐1α and antioxidant enzymes in the liver. (A) Western blotting for antioxidant enzymes (SOD2, catalase, and GPx‐1) and PGC‐1α in liver specimens of bile duct ligation (BDL) mice. The expression of PGC‐1α progressively decreased over time. Of antioxidant enzymes, the expression of SOD2 and GPx‐1 progressively decreased over time, and the expression of catalase decreased after peaking for 2‐3 days after BDL. Relative densities of individual markers had been quantified using ImageJ software and then were normalized to those of β‐actin in each group. Statistical comparison was performed using Mann–Whitney *U*‐test. (B) Immunohistochemical staining for PGC‐1α, SOD2, and Bax after generating BDL. Staining for PGC‐1α and SOD2 progressively decreased after BDL. Contrastingly, Bax staining peaked 3 days after BDL and decreased thereafter. Percentages of immunoreactive areas were measured using NIH ImageJ and expressed as relative values to those in normal livers. Statistical comparison was performed using Kruskal–Wallis test [scale bar, 200 μm (left panels) and 50 μm (right panels)]. Values are presented as mean ± standard deviation of three independent experiments. *P < 0.05. Abbreviations: Bax, Bcl‐2‐like protein 4; GPx‐1α, glutathione peroxidase‐1 alpha; PGC‐1α, peroxisome proliferator‐activated receptor‐γ co‐activator 1α; SOD2, superoxide dismutase 2.

To determine the effects of BDL on the expression of antioxidant enzymes and PGC‐1α in the liver, we determined the expression of these kinds of proteins in the liver specimens of BDL mice using western blot analysis (Fig. 4A). The expression of PGC‐1α progressively decreased over time. Of antioxidant enzymes, the expression of SOD2 and GPx‐1 progressively decreased and the expression of catalase abruptly decreased after temporal elevation over time.

Next, we performed immunohistochemical staining of liver specimens to determine the expression of PGC‐1α, SOD2, and Bax after generation of BDL (Fig. 4B). Similar to the results of western blotting, the areas stained positively for PGC‐1α and SOD2 progressively decreased over time after generation of BDL. By contrast, the staining of areas of liver specimens positive for Bax (a pro‐apoptotic marker) peaked on 3 days after BDL and decreased thereafter.

### Comparing TAA‐induced fibrotic and cholestatic livers in terms of the expression of antioxidant enzymes and PGC‐1α

**Fig. 5:**
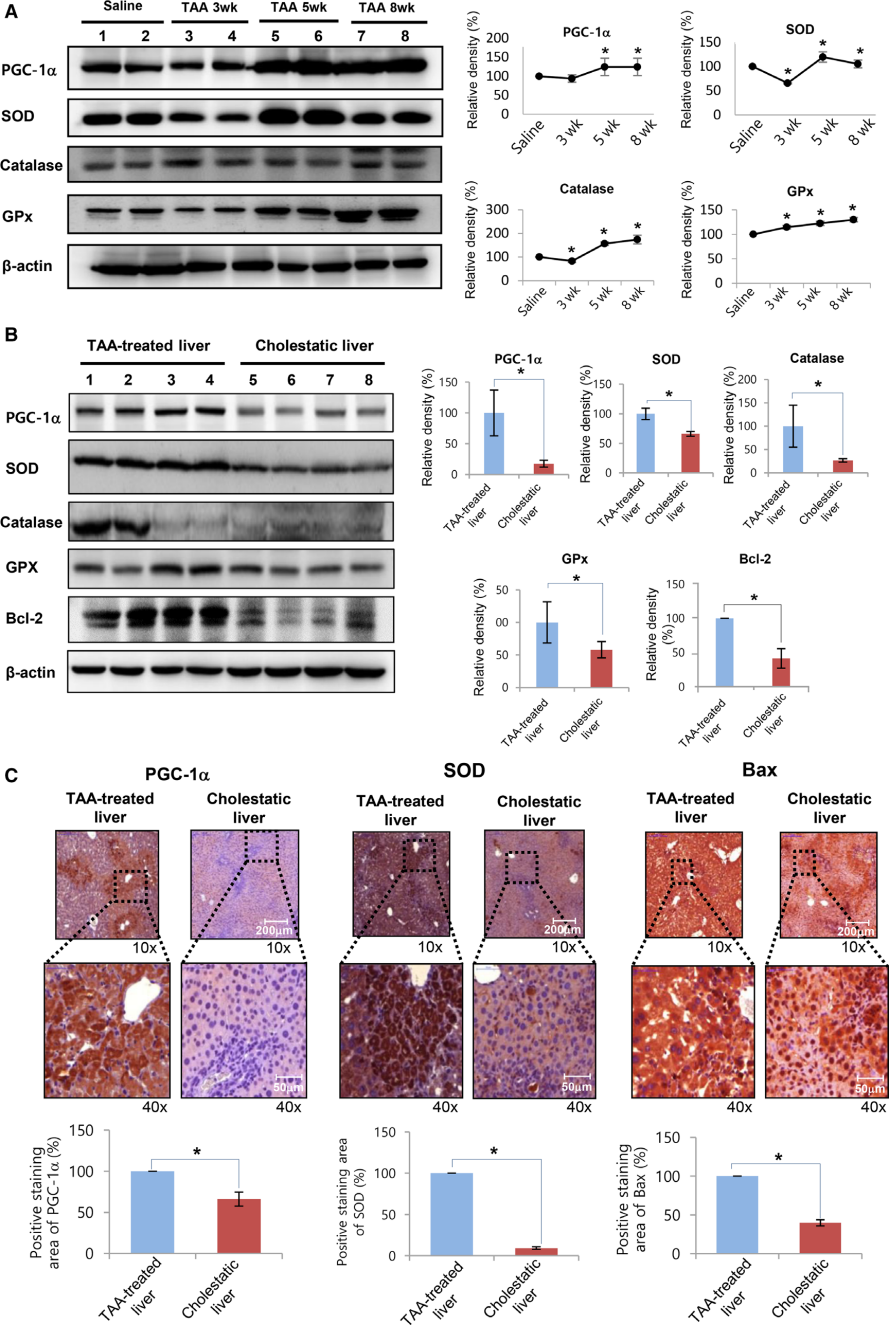
Comparison of the expression of antioxidant enzymes and PGC‐1α between TAA‐treated and cholestatic livers. (A) Western blotting for antioxidant enzymes (SOD2, catalase, and GPx‐1) and PGC‐1α in liver specimens following TAA treatment over time. The expression of antioxidant enzymes and PGC‐1 appeared to be progressively increased over time. (B) Western blotting for antioxidant enzymes and PGC‐1α in both liver fibrosis and obstructive cholestasis. The liver specimens obtained from BDL mice (with cholestatic livers) exhibited significantly lesser expression of antioxidant enzymes (SOD2, catalase, and GPx‐1), PGC‐1α, and Bcl‐2 than that seen in the liver specimens from TAA‐treated mice (with TAA‐induced fibrotic livers). Relative densities of individual markers had been quantified using ImageJ software and then were normalized to those of β‐actin in each group. (C) Immunohistochemical staining for PGC‐1α, SOD2, and Bax in both liver fibrosis and obstructive cholestasis. TAA‐induced fibrotic livers exhibited significantly higher expression of PGC‐1α, SOD2, and Bax than that seen in the cholestatic livers. Percentages of immunoreactive areas were measured using NIH ImageJ and expressed as relative values to those in normal livers. Statistical comparison was performed using Mann–Whitney *U*‐test [scale bar, 200 μm (upper panels) and 50μm (lower panels)]. Values are presented as mean ± standard deviation of three independent experiments. *P < 0.05. Abbreviations: Bax, Bcl‐2‐like protein 4; Bcl‐2, B‐cell lymphoma 2; BDL, bile duct ligation; GPx‐1α, glutathione peroxidase‐1 alpha; PGC‐1α, peroxisome proliferator‐activated receptor‐γ co‐activator 1α; SOD2, superoxide dismutase 2; TAA, thioacetamide.

Clinically, liver fibrosis and cholestasis are two major diseases, ultimately leading to hepatic failure. Thus, we intended to compare the pathogenesis of each disease on the molecular basis. Besides the mouse model of cholestasis, we generated a mouse model of liver fibrosis (*N* = 20) by intraperitoneal injection of TAA (200 mg kg^−1^ body weight) two times a week for 8 weeks. Western blot analyses showed that the expression of antioxidant enzymes (SOD2, catalase, and GPx‐1) and PGC‐1α in liver specimens appeared to be progressively increased over time (Fig. 5A). To compare the pathogenesis of liver fibrosis and obstructive cholestasis, we performed western blotting for antioxidant enzymes and PGC‐1α in both mouse models (Fig. 5B). The liver specimens obtained from BDL mice (cholestatic livers) exhibited significantly reduced expression of antioxidant enzymes (SOD2, catalase, and GPx‐1) and PGC‐1α than that in the liver specimens from TAA‐treated mice (TAA‐induced fibrotic livers) (*P* < 0.05). Additionally, we compared the expression of Bcl‐2 (an anti‐apoptotic marker) in both models. Whereas TAA‐induced fibrotic livers exhibited significantly higher expression of Bcl‐2, cholestatic livers did not.

Next, we compared the expression of PGC‐1α, SOD2, and Bax in both models of TAA‐induced fibrotic and cholestatic livers using immunohistochemical staining (Fig. 5C). Consistent with the results of western blotting, cholestatic livers exhibited significantly lower expression of PGC‐1α, SOD2, and Bax than the cholestatic livers (*P* < 0.05). Taken altogether, our results indicate that cholestatic livers exhibited lower expression of antioxidant enzymes (SOD2, catalase, and GPx‐1) as well as that of pro‐apoptotic proteins (Bax) than TAA‐induced fibrotic livers. In addition, expression of PGC‐1α was found to be significantly reduced in cholestatic livers than TAA‐induced fibrotic livers.

### Comparing TAA‐induced fibrotic and cholestatic livers in terms of the expression of markers for oxidative stress

**Fig. 6:**
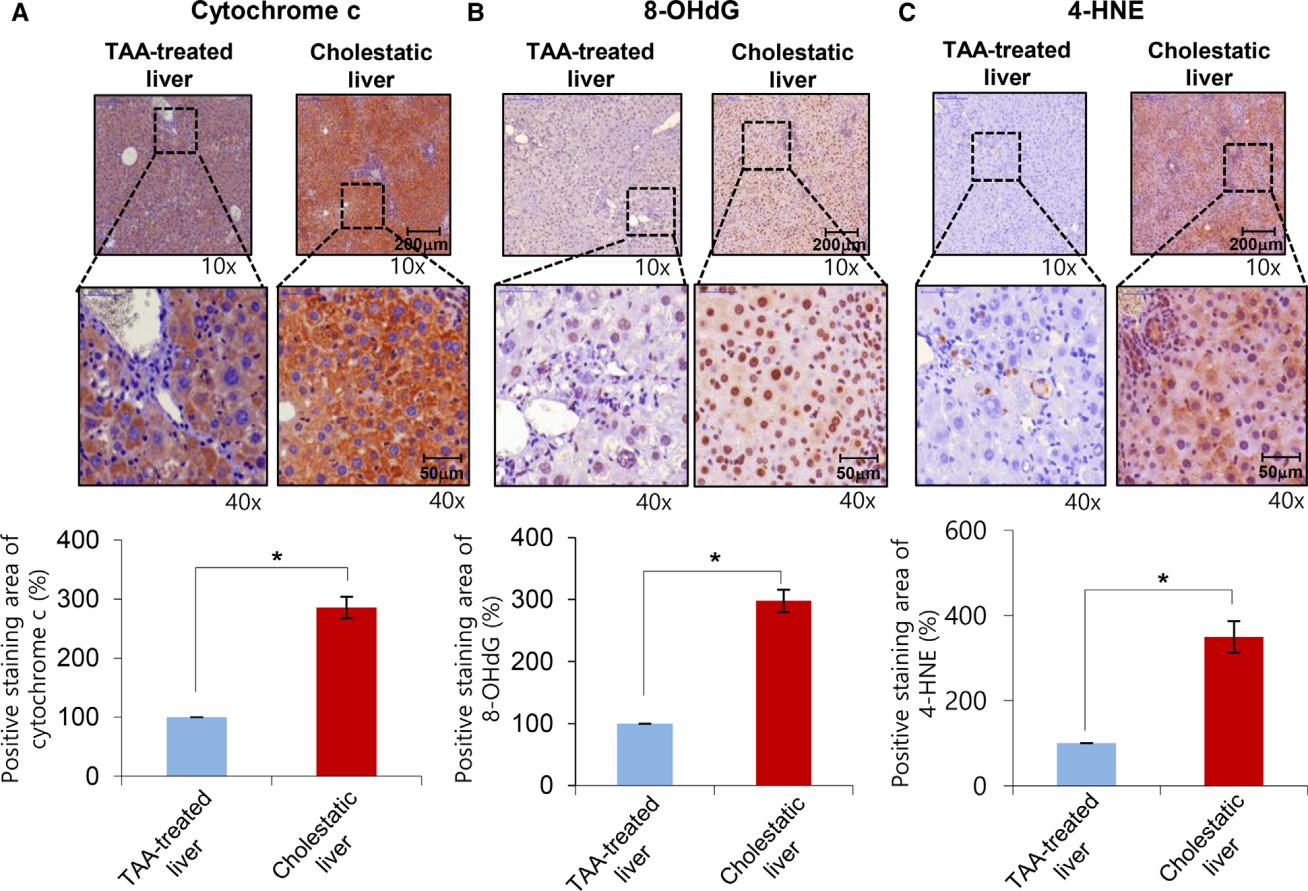
Comparing TAA‐induced fibrotic and cholestatic livers in terms of the expression of markers for oxidative stress. (A) Immunohistochemical staining for cytochrome c in both liver fibrosis and obstructive cholestasis. Cholestatic livers exhibited significantly higher expression of cytochrome c than did fibrotic livers [scale bar, 200μm (upper panels) and 50μm (lower panels)]. (B) Immunohistochemical staining for 8‐OHdG in both liver fibrosis and obstructive cholestasis. Cholestatic livers exhibited significantly higher expression of 8‐OHdG than did fibrotic livers [scale bar, 200 μm (upper panels) and 50μm (lower panels)]. (C) Immunohistochemical staining for 4‐HNE in both liver fibrosis and obstructive cholestasis. Cholestatic livers exhibited significantly higher expression of 4‐HNE than did fibrotic livers. Percentages of immunoreactive areas were measured using NIH ImageJ and expressed as relative values to those in normal livers. Statistical comparison was performed using Mann–Whitney *U*‐test [scale bar, 200 μm (upper panels) and 50 μm (lower panels)]. Values are presented as mean ± standard deviation of three independent experiments. *P < 0.05. Abbreviations: 4‐HNE, 4‐hydroxy‐2‐nonenal; 8‐OHdG, 8‐hydroxy‐2'‐deoxyguanosine; BDL, bile duct ligation; PGC‐1α, peroxisome proliferator‐activated receptor‐γ co‐activator 1α; TAA, thioacetamide

We compared the expression of cytochrome c, 8‐OHdG, and 4‐HNE in both models of TAA‐induced fibrotic and cholestatic livers using immunohistochemical staining. Cytochrome c is known for a marker of mitochondrial damage because the opening of the MPT pore can promote release of cytochrome c [14]. Immunohistochemical stains of cytochrome c indicated that cholestatic livers exhibited significantly higher expression of cytochrome c than did fibrotic livers (*P* < 0.05) (Fig. 6A). Subsequently, immunohistochemical stains for the markers for oxidative stress (8‐OHdG and 4‐HNE) were utilized for determining oxidative stress in each group. Cholestatic livers exhibited higher expression of 8‐OHdG and 4‐HNE than did fibrotic livers (*P* < 0.05) (Fig. 6B,C). Taken together, it appears that cholestatic livers have higher rates of MPT pore opening and higher levels of oxidative stress due to mitochondrial damage than do fibrotic livers.

### Comparing biochemical parameters reflecting hepatic function between TAA‐induced fibrotic and cholestatic livers

**Fig. 7:**
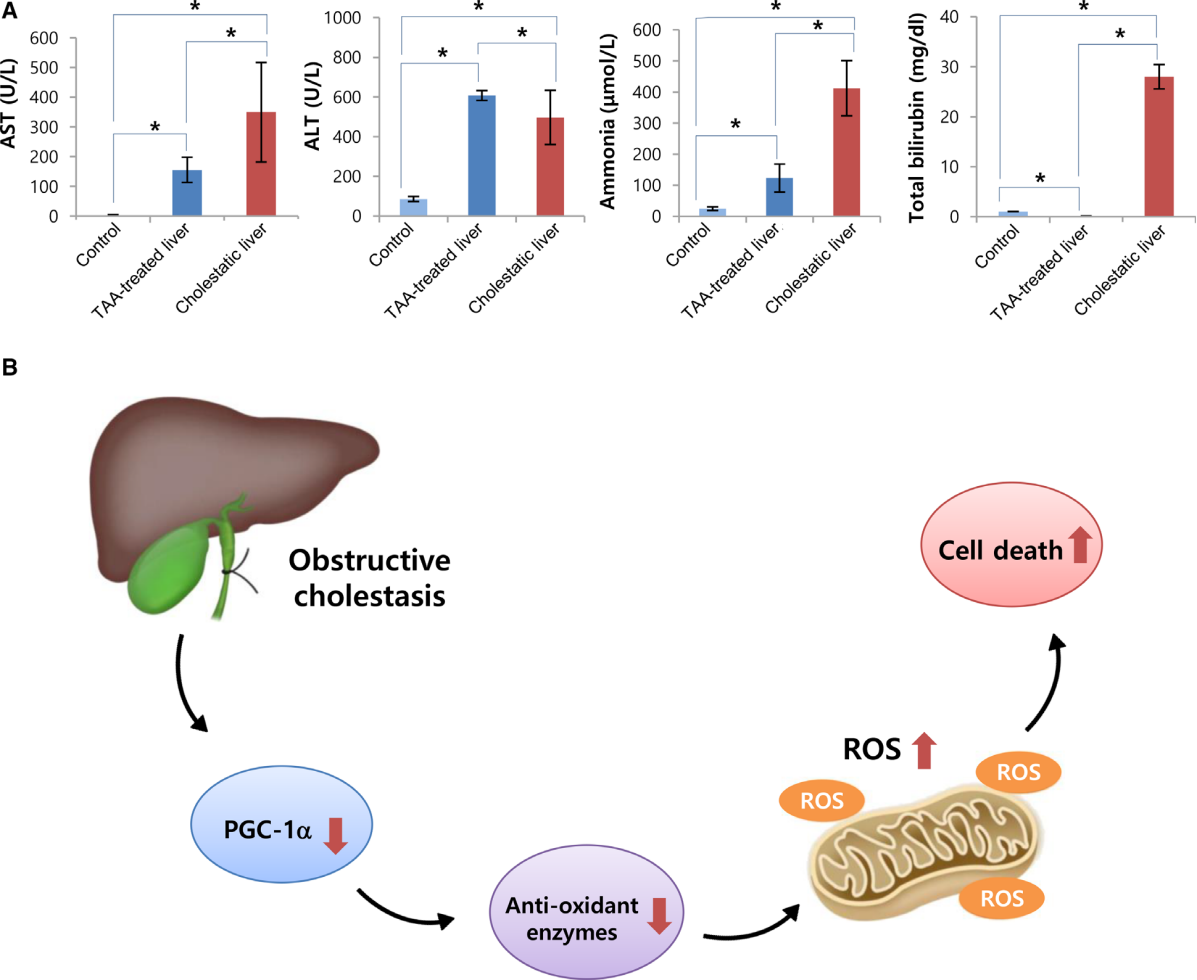
Comparison of biochemical parameters reflecting hepatic function between TAA‐induced fibrotic and cholestatic livers. (A) Serology tests comparing mice with obstructive cholestasis and TAA‐induced fibrosis. Mice with obstructive cholestasis exhibited significantly higher expression of all biochemical parameters than that seen in mice with TAA‐induced fibrotic livers. Statistical comparison was performed using Kruskal–Wallis test. (B) Proposed mechanism leading to cell death in the cholestatic liver disease. Values are presented as mean ± standard deviation of three independent experiments. *P < 0.05. Abbreviations: ALT, alanine transaminase; AST, aspartate transaminase; BDL, bile duct ligation; TAA, thioacetamide.

Finally, we compared the serum concentration of biochemical parameters (AST, ALT, ammonia, and total bilirubin) reflecting hepatic function between the two models (Fig. 7A). The mice with cholestatic livers exhibited significantly higher expression of all biochemical parameters than that in mice with TAA‐induced fibrotic livers (all *P* < 0.05). The difference was most prominent in the comparison of the serum levels of total bilirubin. Taken altogether, we illustrated the possible mechanism of cell damage following obstructive cholestasis in Fig. 7B.

## Discussion

We herein investigated the pathogenesis of obstructive cholestatic diseases, especially in relation to the role of mitochondria. Our results were largely based on our *in vivo* experiments of mice with BDL. Consistent with previous studies, cholestatic mice showed the lower expression of antioxidant enzymes, such as SOD2, catalase, and GPx‐1, as well as PGC‐1α. Subsequently, BDL mice (with cholestatic livers) showed lower expression of antioxidant enzymes as well as PGC‐1α than the mice with TAA‐induced liver fibrosis. It was well known that overexpression of PGC‐1α increases mitochondrial antioxidant enzyme expression, and vice versa [11, 12]. Thus, we could conclude that obstructive cholestasis accompanies the lower expression of PGC‐1α, which leads to decreased expression of mitochondrial antioxidant enzymes, rendering cholestatic livers vulnerable to ROS‐induced cell death.

In this study, we reaffirmed that mitochondrial dysfunction is principally involved in the pathogenesis of cholestatic liver diseases. One of the most detrimental factors of obstructive cholestasis is the intrahepatic accumulation of hydrophobic bile salts, which are particularly hepatotoxic. Numerous studies indicate that mitochondria are a primary target of toxic bile salts [6, 15, 16, 17, 18, 19, 20, 21, 22]. Hydrophobic bile acids increase generation of ROS in hepatic mitochondria of rats [4, 23], lead to depletion of antioxidant defenses [24, 25], and induce mitochondrial permeability transition (MPT), a critical intracellular trigger of cell death in hepatocytes [16, 19, 22, 26, 27]. MPT is characterized by an increase in permeability of the inner membrane of mitochondria to low‐molecular‐weight solutes, leading to depolarization of the mitochondrial membrane, mitochondrial calcium release, and inhibition of oxidative phosphorylation [27].

Opening of the MPT pore causes mitochondrial swelling, depletion of the mitochondrial membrane potential, and reduction of ATP production, all of which ultimately lead to necrotic cell death [26]. It was found that blocking the MPT prevented both apoptotic and necrotic hepatocyte death stimulated by bile acids [16, 20, 22]. Our results suggest that cholestatic liver diseases could be ameliorated by reduction of oxidative stress. Indeed, bile acid‐induced necrotic cell death of hepatocytes was inhibited by antioxidant enzymes, such as catalase [15], superoxide dismutase [15], oxypurinol [21], a lazaroid [28], and idebenone [20]. The significance of oxidative stress in the pathogenesis of cholestatic liver diseases is also demonstrated by the report revealing that the antioxidant enzyme status in isolated rat hepatocytes determines whether the liver progresses to bile acid‐induced necrosis [17].

In this study, contrary to TAA‐induced liver fibrosis, which showed higher expression levels of PGC‐1α, cholestatic livers showed lower expression of PGC‐1α. PGC‐1α acts as a transcriptional co‐activator that potentiates numerous transcription factors whose functions include mitochondrial biogenesis, adaptive thermogenesis, glucose/fatty acid metabolism, and fiber type switching in skeletal muscle [29]. Particularly, there is clear‐cut causal relationship between PGC‐1α and antioxidant enzymes; previous literature showed that overexpressing PGC‐1α leads to upregulation of antioxidant enzymes, thereby protects cells from ROS [11, 30, 31]. Endothelial cells overexpressing PGC‐1α showed that reduced accumulation of ROS, increased mitochondrial membrane potential, and reduced apoptotic cell death [11]. In addition, downregulation of PGC‐1α levels by siRNA reduces the expression of mitochondrial antioxidant proteins. In this study, we also found that overexpression of PGC‐1α led to the upregulation of antioxidant enzymes.

We also found that cholestatic livers showed lower expression of pro‐apoptotic markers (Bax and Bcl‐2) than TAA‐induced fibrotic livers. An essential debate on the mechanisms of cell death in cholestatic livers is whether the cell death is caused by apoptosis or necrosis [32]. Whereas apoptosis is characterized by cellular shrinking, caspase activation, and DNA fragmentation, necrosis is characterized by cellular swelling, membrane blebbing, DNA fragmentation, and release of cellular components [33]. Our results support that necrosis could be the principal causative factor for cholestatic liver diseases. Hepatotoxic bile acids hardly reach the concentrations that might directly cause cell death in cholestatic livers. Instead, they can easily trigger inflammatory mediator formation, which initiates an inflammatory response and necrotic cell death caused by neutrophils through oxidant stress [34].

In conclusion, we found that obstructive cholestasis shows decreased expression of PGC‐1α, which leads to decreased expression of mitochondrial antioxidant enzymes, rendering cholestatic livers vulnerable to ROS‐induced cell death. The results of the current study provide a scientific basis for any measures to reduce oxidative stress to overcome cholestatic liver diseases.

## Author contribution

KSJ planned the study, interpreted the data, and prepared the manuscript. PJH wrote the manuscript and principally performed in vivo experiments. KOH and HHE performed in vitro experiments. KBJ, CHJ, LSC, KKH, YYK, LTY, and AJ performed a number of experiments as a team and analyzed the data. All authors read and approved the manuscript.

## Conflict of interest

The authors declare no conflict of interest.

## Data Availability

The datasets generated during and/or analyzed during the current study are available from the corresponding author on reasonable request.

## References

[1] Scott‐Conner CE and Grogan JB (1994) The pathophysiology of biliary obstruction and its effect on phagocytic and immune function. J Surg Res 57, 316–336. 8028341 10.1006/jsre.1994.1151

[2] de Buy Maillette , Wenniger L and Beuers U (2010) Bile salts and cholestasis. Dig Liver Dis 42, 409–418. 20434968 10.1016/j.dld.2010.03.015

[3] Rolo AP , Palmeira CM and Wallace KB (2003) Mitochondrially mediated synergistic cell killing by bile acids. Biochim Biophys Acta 1637, 127–132. 12527417 10.1016/s0925-4439(02)00224-7

[4] Sokol RJ , Dahl R , Devereaux MW , Yerushalmi B , Kobak GE and Gumpricht E (2005) Human hepatic mitochondria generate reactive oxygen species and undergo the permeability transition in response to hydrophobic bile acids. J Pediatr Gastroenterol Nutr 41, 235–243. 16056106 10.1097/01.mpg.0000170600.80640.88

[5] Palmeira CM and Rolo AP (2004) Mitochondrially‐mediated toxicity of bile acids. Toxicology 203, 1–15. 15363577 10.1016/j.tox.2004.06.001

[6] Sokol RJ , Winklhofer‐Roob BM , Devereaux MW and McKim JM Jr (1995) Generation of hydroperoxides in isolated rat hepatocytes and hepatic mitochondria exposed to hydrophobic bile acids. Gastroenterology 109, 1249–1256. 7557092 10.1016/0016-5085(95)90585-5

[7] Arany Z , He H , Lin J , Hoyer K , Handschin C , Toka O , Ahmad F , Matsui T , Chin S , Wu PH *et al* (2005) Transcriptional coactivator PGC‐1 alpha controls the energy state and contractile function of cardiac muscle. Cell Metab 1, 259–271. 16054070 10.1016/j.cmet.2005.03.002

[8] Finck BN and Kelly DP (2006) PGC‐1 coactivators: inducible regulators of energy metabolism in health and disease. J Clin Invest 116, 615–622. 16511594 10.1172/JCI27794 PMC1386111

[9] Kelly DP and Scarpulla RC (2004) Transcriptional regulatory circuits controlling mitochondrial biogenesis and function. Genes Dev 18, 357–368. 15004004 10.1101/gad.1177604

[10] Wu Z , Puigserver P , Andersson U , Zhang C , Adelmant G , Mootha V , Troy A , Cinti S , Lowell B , Scarpulla RC *et al* (1999) Mechanisms controlling mitochondrial biogenesis and respiration through the thermogenic coactivator PGC‐1. Cell 98, 115–124. 10412986 10.1016/S0092-8674(00)80611-X

[11] Valle I , Alvarez‐Barrientos A , Arza E , Lamas S and Monsalve M (2005) PGC‐1alpha regulates the mitochondrial antioxidant defense system in vascular endothelial cells. Cardiovasc Res 66, 562–573. 15914121 10.1016/j.cardiores.2005.01.026

[12] St‐Pierre J , Drori S , Uldry M , Silvaggi JM , Rhee J , Jager S , Handschin C , Zheng K , Lin J , Yang W *et al* (2006) Suppression of reactive oxygen species and neurodegeneration by the PGC‐1 transcriptional coactivators. Cell 127, 397–408. 17055439 10.1016/j.cell.2006.09.024

[13] Tag CG , Weiskirchen S , Hittatiya K , Tacke F , Tolba RH and Weiskirchen R (2015) Induction of experimental obstructive cholestasis in mice. Lab Anim 49, 70–80. 25835740 10.1177/0023677214567748

[14] Buki A , Okonkwo DO , Wang KK and Povlishock JT (2000) Cytochrome c release and caspase activation in traumatic axonal injury. J Neurosci 20, 2825–2834. 10751434 10.1523/JNEUROSCI.20-08-02825.2000 PMC6772193

[15] Sokol RJ , Devereaux M , Khandwala R and O'Brien K (1993) Evidence for involvement of oxygen free radicals in bile acid toxicity to isolated rat hepatocytes. Hepatology 17, 869–881. 8387948

[16] Yerushalmi B , Dahl R , Devereaux MW , Gumpricht E and Sokol RJ (2001) Bile acid‐induced rat hepatocyte apoptosis is inhibited by antioxidants and blockers of the mitochondrial permeability transition. Hepatology 33, 616–626. 11230742 10.1053/jhep.2001.22702

[17] Gumpricht E , Devereaux MW , Dahl RH and Sokol RJ (2000) Glutathione status of isolated rat hepatocytes affects bile acid‐induced cellular necrosis but not apoptosis. Toxicol Appl Pharmacol 164, 102–111. 10739750 10.1006/taap.2000.8894

[18] Granato A , Gores G , Vilei MT , Tolando R , Ferraresso C and Muraca M (2003) Bilirubin inhibits bile acid induced apoptosis in rat hepatocytes. Gut 52, 1774–1778. 14633961 10.1136/gut.52.12.1774 PMC1773880

[19] Rodrigues CM , Fan G , Wong PY , Kren BT and Steer CJ (1998) Ursodeoxycholic acid may inhibit deoxycholic acid‐induced apoptosis by modulating mitochondrial transmembrane potential and reactive oxygen species production. Mol Med 4, 165–178. 9562975 PMC2230355

[20] Shivaram KN , Winklhofer‐Roob BM , Straka MS , Devereaux MW , Everson G , Mierau GW and Sokol RJ (1998) The effect of idebenone, a coenzyme Q analogue, on hydrophobic bile acid toxicity to isolated rat hepatocytes and hepatic mitochondria. Free Radic Biol Med 25, 480–492. 9741584 10.1016/s0891-5849(98)00077-x

[21] Sokol RJ , Devereaux M and Khandwala RA (1991) Effect of dietary lipid and vitamin E on mitochondrial lipid peroxidation and hepatic injury in the bile duct‐ligated rat. J Lipid Res 32, 1349–1357. 1770317

[22] Sokol RJ , Straka MS , Dahl R , Devereaux MW , Yerushalmi B , Gumpricht E , Elkins N and Everson G (2001) Role of oxidant stress in the permeability transition induced in rat hepatic mitochondria by hydrophobic bile acids. Pediatr Res 49, 519–531. 11264436 10.1203/00006450-200104000-00014

[23] Gumpricht E , Devereaux MW , Dahl R , Soden JS , Sparagna GC , Leonard SW , Traber MG and Sokol RJ (2008) Resistance of young rat hepatic mitochondria to bile acid‐induced permeability transition: potential role of alpha‐tocopherol. Pediatr Res 64, 498–504. 18596569 10.1203/PDR.0b013e3181841ee1 PMC2651029

[24] Serviddio G , Pereda J , Pallardo FV , Carretero J , Borras C , Cutrin J , Vendemiale G , Poli G , Vina J and Sastre J (2004) Ursodeoxycholic acid protects against secondary biliary cirrhosis in rats by preventing mitochondrial oxidative stress. Hepatology 39, 711–720. 14999689 10.1002/hep.20101

[25] Krahenbuhl S , Talos C , Lauterburg BH and Reichen J (1995) Reduced antioxidative capacity in liver mitochondria from bile duct ligated rats. Hepatology 22, 607–612. 7635430 10.1002/hep.1840220234

[26] Kim JS , He L and Lemasters JJ (2003) Mitochondrial permeability transition: a common pathway to necrosis and apoptosis. Biochem Biophys Res Commun 304, 463–470. 12729580 10.1016/s0006-291x(03)00618-1

[27] Zoratti M and Szabo I (1995) The mitochondrial permeability transition. Biochim Biophys Acta 1241, 139–176. 7640294 10.1016/0304-4157(95)00003-a

[28] Patel T and Gores GJ (1997) Inhibition of bile‐salt‐induced hepatocyte apoptosis by the antioxidant lazaroid U83836E. Toxicol Appl Pharmacol 142, 116–122. 9007040 10.1006/taap.1996.8031

[29] Liang H and Ward WF (2006) PGC‐1alpha: a key regulator of energy metabolism. Adv Physiol Educ 30, 145–151. 17108241 10.1152/advan.00052.2006

[30] Bellafante E , Morgano A , Salvatore L , Murzilli S , Di Tullio G , D'Orazio A , Latorre D , Villani G and Moschetta A (2014) PGC‐1beta promotes enterocyte lifespan and tumorigenesis in the intestine. Proc Natl Acad Sci U S A 111, E4523–4531. 25288742 10.1073/pnas.1415279111 PMC4210309

[31] Nijland PG , Witte ME , van het Hof B , van der Pol S , Bauer J , Lassmann H , van der Valk P , de Vries HE and van Horssen J (2014) Astroglial PGC‐1alpha increases mitochondrial antioxidant capacity and suppresses inflammation: implications for multiple sclerosis. Acta Neuropathol Commun 2, 170. 25492529 10.1186/s40478-014-0170-2 PMC4268800

[32] Jaeschke H , Gujral JS and Bajt ML (2004) Apoptosis and necrosis in liver disease. Liver Int 24, 85–89. 15078470 10.1111/j.1478-3231.2004.0906.x

[33] Jaeschke H and Lemasters JJ (2003) Apoptosis versus oncotic necrosis in hepatic ischemia/reperfusion injury. Gastroenterology 125, 1246–1257. 14517806 10.1016/s0016-5085(03)01209-5

[34] Woolbright BL and Jaeschke H (2012) Novel insight into mechanisms of cholestatic liver injury. World J Gastroenterol 18, 4985–4993. 23049206 10.3748/wjg.v18.i36.4985 PMC3460324

